# Precision, accuracy, and reliability of a threshold hunting method for transcranial magnetic stimulation

**DOI:** 10.1016/j.cnp.2025.12.008

**Published:** 2025-12-24

**Authors:** Yuichiro Shirota, Juuri Otsuka, Masashi Hamada

**Affiliations:** a Department of Clinical Laboratory, the University of Tokyo Hospital, Tokyo, Japan; b Department of Neurology, Graduate School of Medicine, The University of Tokyo, Tokyo, Japan

**Keywords:** Transcranial magnetic stimulation, Resting motor threshold, Automation, Maximum likelihood estimation, Adaptive method, Precision, Accuracy

## Abstract

**Objective:**

To investigate precision, accuracy, and reliability of a threshold hunting method to estimate resting motor threshold (RMT) using parameter estimation by sequential testing and maximum likelihood estimation (PEST-MLE).

**Methods:**

In 53 healthy participants, single pulse transcranial magnetic stimulation was delivered to estimate RMT with cutoff values of 0.05 mV and 0.2 mV. RMT was inferred from 30-trial PEST-MLE algorithm for at maximum two days with two estimation sessions per day, comprising Sessions 1 to 4. Precision and accuracy were assessed within each session. Repeatability was assessed using intraclass correlation coefficient (ICC), reproducibility coefficient (RC), and standard error of measurement (SEM).

**Results:**

For both 0.05 mV and 0.2 mV, 18 trials were needed to have good accuracy. ICC greater than 0.8 was achieved for within-day comparison but the ICC of between-day comparison was lower. RC and SEM were around 10 % and 5 %, respectively.

**Conclusions:**

At 18th trial the estimations were converged in terms of precision and accuracy, and good reliability was achieved at that stage.

**Significance:**

RMT estimation with the PEST-MLE is a rapid and reliable approach that can be implemented for future clinical and scientific studies.

## Highlights

• Adaptive method estimated motor threshold precisely and accurately in 18 trials.
• Within-day reliability was good: intraclass correlation coefficients were >0.80.
• Reproducibility coefficient was around 10 % with the standard error around 5 %

## 1 Introduction

Estimation of the resting motor threshold (RMT) is important for clinical and research applications of transcranial magnetic stimulation (TMS). In fact, RMT serves as the reference point for determining stimulus intensities in studies of cortical excitability and plasticity, and in therapeutic protocols such as repetitive TMS for depression, stroke, and other disorders. The motor threshold is typically defined as the minimum stimulus intensity which elicits a motor evoked potential (MEP) exceeding a predefined amplitude in more than half of trials (Rossini et al., 1994). For RMT, the cut-off amplitude is usually set as 0.05 mV. Accurate and reliable determination of RMT is crucially important because mistaken estimation can affect both safety and efficacy directly in clinical and experimental settings.

Conventionally, RMT has been estimated using the so-called relative frequency method (Rossini et al., 1994), by which stimuli with a certain intensity are delivered, typically 10 times. Then, if MEPs greater than 0.05 mV are obtained more than five times, a slightly lower intensity would be tested; otherwise slightly higher intensity would be tested. Although it is used widely, this procedure is time-consuming. Moreover, it might be influenced by operator decisions.

Adaptive methods have been developed to overcome these limitations. The adaptive methods assume a sigmoid relation between TMS intensity and the probability of obtaining MEP amplitude greater than a certain value, e.g. 0.05 mV. Given this relation, the RMT is estimated iteratively: if a TMS intensity evoked an MEP, then the next trial would test lower intensity to ascertain whether it “failed” or still “succeeded” in evoking an MEP. An implementation of this procedure is proposed using parameter estimation by sequential testing (PEST) and maximum likelihood estimation (MLE) (Awiszus, 2003). The latest guideline described that both the relative frequency methods and the adaptive methods are acceptable for clinical and research use, but it also stated that adaptive methods might provide faster and more accurate estimation (Rossini et al., 2015).

Despite this potential, evidence related to the precision, accuracy, and test–retest reliability of adaptive methods remains limited. Reproducibility of an adaptive method other than the PEST-MLE was reported using intraclass correlation coefficient (ICC)-based analyses (Samusyte et al., 2018). By contrast, more recent studies (Tankisi et al., 2022, 2021) developed more sophisticated versions of the threshold-tracking approach, primarily emphasizing protocol shortening by reducing the number of stimuli per condition and often without reporting formal reliability indices. These approaches differ methodologically from the PEST–MLE algorithm (Awiszus, 2003). For the present study, we adopted a complementary approach by evaluating the stability of estimates (precision) and their closeness to a reference threshold (accuracy) separately, with multiple reliability indices including ICC, the reproducibility coefficient (RC), and the standard error of measurement (SEM). Although the reference threshold was defined pragmatically as the mean threshold estimate of the last trials in this study, this operational definition served as a consistent benchmark within our analyses, allowing systematic investigation of how many trials must be conducted to obtain both precise and accurate estimates.

Therefore, this study was conducted to elucidate the precision, accuracy, and test–retest reliability of the PEST-MLE algorithm when used to estimate RMT in healthy adults, and to ascertain the number of trials necessary to achieve stable convergence under typical experiment conditions.

## 2 Methods

### 2.1 Participants and ethics statements

For this study, 53 healthy volunteers were recruited via advertisement. The inclusion criteria were healthy adults with no neurological or psychiatric medical history, who were taking no medication that might affect central nervous system function. Contraindications for TMS were screened, using a questionnaire similar to a published one (Rossi et al., 2011), to exclude persons with metal implants near the head or with history of seizure or epilepsy, pregnant women, and persons with other contraindications. No strict regulations were imposed on participants regarding sleep or caffeine intake; however, they were instructed to maintain their usual lifestyle as much as possible. The time of day (morning or afternoon) was kept constant for each participant.

Written informed consent was obtained from all participants. The local ethics committee approved the study protocol (No. 11936-(2)).

### 2.2 Recording and stimulation

Surface electromyography (EMG) was recorded from the right first dorsal interosseous muscle using Ag/AgCl electrodes placed according to the belly tendon montage. After the signals were amplified and band-pass filtered between 2 and 3000 Hz (Neuropack S1; Nihon-Koden Co. Ltd., Tokyo, Japan), they were digitized at 5 kHz with the CED Power 1401 mk II (Cambridge Electronic Design Ltd., Cambridge, UK) and were sent to a computer for assessing MEP amplitude using the script described below.

TMS was delivered using a Magstim200^2^ stimulator with a 70-mm figure-of-eight coil (The Magstim Company Ltd., Carmarthenshire, UK). For the paired-pulse paradigm, which was not included in this study, two stimulators were connected with a Bistim module (The Magstim Company Ltd.); only one of them was used for this study. The TMS coil was positioned backward 45-degree away from midline to produce a posterior-to-anterior current across central sulcus in the cortex. For each experiment, the participant’s hot spot was searched manually by delivering a slightly suprathreshold stimuli over different points near a hand motor area. The search started from a point 5 cm lateral to the left from the vertex. The hand-held TMS coil was moved in approximately 1-cm steps until the point was found where the largest MEP was produced stably (Rossini et al., 2015). The basic approach in the determination of the hot spot was in line with a previous study (Meincke et al., 2016); namely, the hot spot was identified through manual probing of nearby scalp sites while adjusting intensity as needed to evoke reliable MEPs (the starting intensity was typically between 40 and 50 % MSO), followed by confirmation that neighboring positions yielded weaker responses. Once the spot was identified, the site was marked with a pen to facilitate coil repositioning. The stimulator communicated with the CED Power 1401 mk II and a computer with the Signal software (both Cambridge Electronic Design Ltd., Cambridge, UK) using a custom-made cable (Miyuki Giken Co. Ltd., Tokyo, Japan), so that depending on the MEP amplitude the next stimulus intensity was changed automatically in the later threshold hunting procedure. For determination of the RMT, two target MEP amplitudes were selected: 0.05 mV as a usual value for RMT; and 0.2 mV, which is often used for threshold tracking methods (Menon et al., 2015, Samusyte et al., 2018, Tankisi et al., 2021, Vucic et al., 2006). They are designated respectively as RMT0.05 and RMT0.2 hereinafter.

### 2.3 Implementation of threshold hunting

**Fig. 1:**
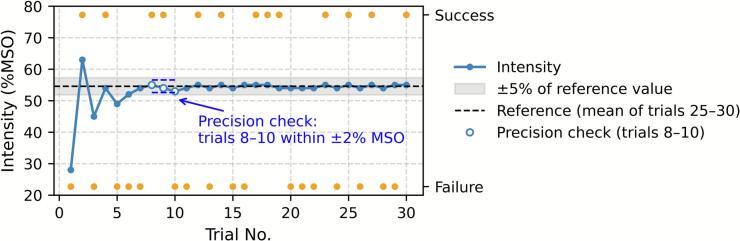
Example of a single session. Blue dots with a line indicate the tested intensity for each trial. Dots on the Success or Failure line indicate whether the trial evoked MEPs larger than the target amplitude (0.05 mV in this case) or not. The estimated RMT0.05 for this session was 55 % MSO. The shaded gray band represents the ±5 % range around the reference value, which was defined as the mean intensity from trials 25–30 in this session (54.6 % MSO in this case). The accuracy criterion was first met at trial 4, but not satisfied at trial 5, then maintained from trial 6 until the end. An example of precision evaluation is illustrated for trials 8–10 with open blue circles. The estimated intensities were checked to fall within ±2 % MSO. In this example, the estimates are 55, 54, and 53 % MSO, and are therefore considered precise at trial 10. MSO, maximum stimulator output. (For interpretation of the references to colour in this figure legend, the reader is referred to the web version of this article.)

The threshold hunting algorithm, which was based on the original report (Awiszus, 2003), was implemented using the Signal Script (Cambridge Electronic Design Ltd., Cambridge, UK). Assuming that stimulation with 0 % maximum stimulator output (%MSO) would not evoke MEP and assuming that with 100 %MSO would, the first trial started with intensity that was chosen randomly from the range of 20 %MSO – 60 %MSO. Given the cumulative Gaussian function representing the probability of obtaining an MEP larger than the target amplitude, the next estimate for RMT was calculated to minimize the log likelihood function using the Levenberg–Marquardt algorithm (Awiszus, 2003). In all, 30 trials were conducted for one condition. Trials showing voluntary EMG activity exceeding 0.05 mV peak-to-peak amplitude during the 65 ms period preceding the stimulus were automatically rejected online. The MEP amplitude was defined as the peak-to-peak value within the 15–40 ms time window after the stimulus. Fig. 1 exhibits an example for one session.

### 2.4 Experiment procedures

Each participant was evaluated with stimuli four times at most: two times on the first day (Sessions 1 and 2), and the other two times on the second day (Sessions 3 and 4). The two experiment days had at least a one week intervening period. After hot spot determination, RMT0.05 was estimated first. Then after some break, if necessary, RMT0.2 was investigated. This experiment session was repeated twice on a single day, with an interval of approximately 5 min, during which the EMG electrodes and other set-ups were left as they were while the TMS coil was taken off from the head. Comparison between Sessions 1 and 2 revealed the within-day reliability. Comparison between Sessions 1 and 3 revealed the between-day reliability. Results of Session 4 were used for assessing precision and accuracy as described below.

### 2.5 Data analysis

Obtained results were analyzed from three points of view: precision, accuracy, and reliability. Indices of precision and accuracy were used to judge at how many trials the session converged. Measures of reliability were calculated to estimate within-day and between-day reliability.

Precision represents the degree to which repeated measurements yield the same values (Doumas, 1997). To estimate the sequential precision of the threshold hunting method, we evaluated whether the estimates from the last three trials were within ±2 % MSO. For example, to assess the precision at the tenth trial, the estimates obtained at the eighth, ninth, and tenth trials were compared (Fig. 1). If the three values were within ±2 % MSO, the precision at that trial was regarded as acceptable. For each measurement session, we determined the trial number at which the precision first reached an acceptable level and maintained thereafter. Since either +1 % MSO or − 1 % MSO change can be acceptable at any trial until a very later stage, ± 2 % was regarded as the minimal value exceeding the fluctuation. Accuracy refers to the degree to which a measurement result corresponds to the true value (Doumas, 1997). To estimate the sequential accuracy of the PEST-MLE threshold-hunting method, we evaluated whether the estimate at each trial was within ±5 % of the reference value, a range corresponding to a 0.05 tolerance level, defined as the mean intensity estimate from trials 25–30 conducted in the same session (Fig. 1). For both indices, we identified the final series of consecutive trials that satisfied the predefined criterion for the remainder of the session. Convergence was defined as the first trial in that series. In practice, once this point was reached, the estimates typically remained stable and continued to meet the criterion through the end of the session. Although this definition might be considered somewhat conservative given the inherent variability of MEPs, it provided a consistent operational criterion for convergence in the present study (*see* Results). Results from Sessions 1–4 were pooled. The first trial at which the convergence criterion was satisfied in at least 95 % of the sessions is reported in the *Results* section.

To investigate test–retest reliability between sessions, we calculated the intraclass correlation coefficient (ICC), the reproducibility coefficient (RC), and the standard error of measurement (SEM). Within-day reliability was tested by comparing Session 1 and Session 2. Between-day reliability was estimated with comparison between Session 1 and Session 3. ICC was computed as ICC(2,1) according to a two-way random-effects model with absolute agreement and single measurements (Shrout and Fleiss, 1979). This coefficient reflects the consistency of threshold estimates across sessions for the same subjects, with values closer to 1.0 indicating excellent reliability. For each trial number (1–30), ICC(2,1) was calculated using the estimates from two sessions. The first trial number at which ICC exceeded 0.80 was defined as the convergence point. RC was calculated based on the within-subject standard deviation *Sw* (Bland and Altman, 1986). Specifically, *Sw* was estimated as the square root of the sum of squared differences between the two sessions divided by twice the number of subjects; also, RC was defined as 1.96 × √2 × *Sw*. RC represents the 95 % limits within which the difference between two measurements from the same subject is expected to lie, with smaller RC indicating better reproducibility. The standard error of measurement (SEM) was derived as SEM = SD × √(1 – ICC), where SD is the between-subject standard deviation (Weir, 2005). SEM provides an estimate of measurement error expressed in the same units as the threshold intensity.

## 3 Results

For the 53 participants, 185 sessions were completed. Session 1 and Session 2 included all participants, but because of errors in data saving, recordings from three participants in Session 2 were lost. The reliability assessment for this comparison was conducted using the remaining 50 participants. They were 26 men and 24 women, and the mean age was 23.7 years (range: 19–50 years). All were right-handed according to the Edinburgh Handedness Inventory (Oldfield, 1971); the mean lateralized quotient was 96.5 (range: 66.7–100). Results of Sessions 3 and 4 were obtained from 41 participants. The between-day reproducibility was inferred using the 41 datasets. In six RMT0.2 sessions (3.2 %), the RMT exceeded 100 % MSO. The RMT estimates for these sessions were defined as 100 % MSO.

### 3.1 Within-day and between-day comparison

**Fig. 2:**
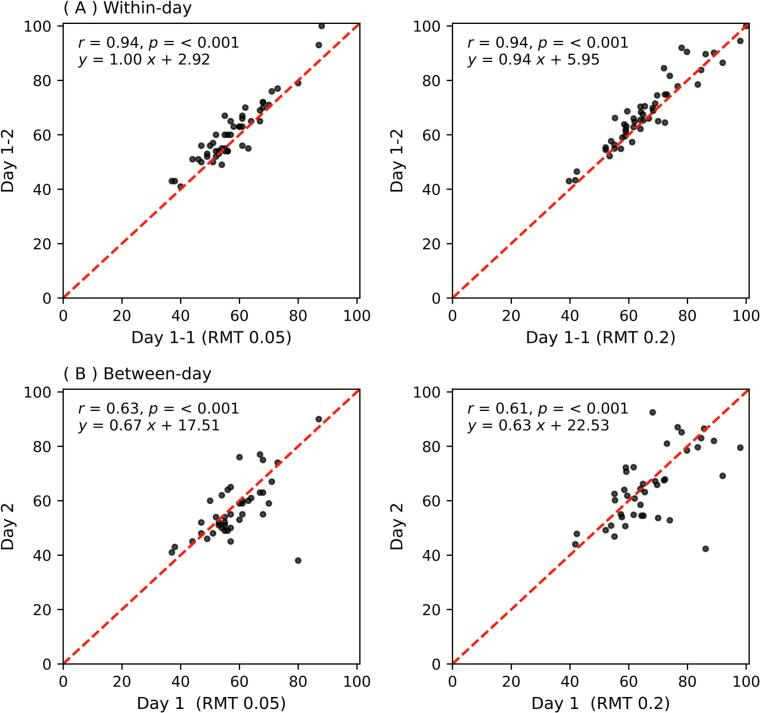
Scatter plots showing (A) within-day and (B) between-day correlations of the RMT estimates. For both (A) and (B), the left panels are for RMT0.05. The right panels are for RMT0.2. Red dashed lines represent equality of the two measurements. RMT, resting motor threshold. (For interpretation of the references to colour in this figure legend, the reader is referred to the web version of this article.)

**Fig. 3:**
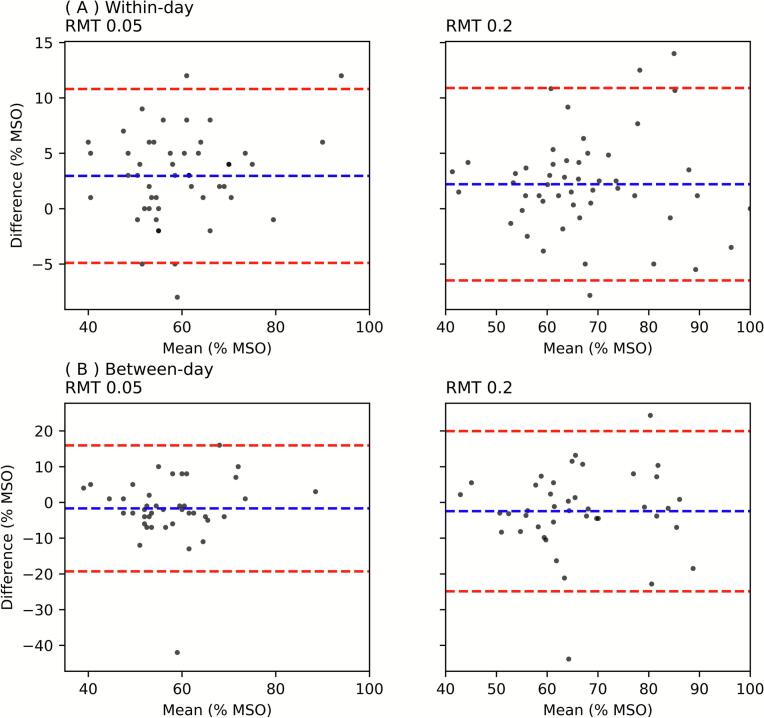
Bland-Altman plots. Using the same data as Fig. 2, Bland-Altman plots are depicted. For both (A) and (B), the left panels are for RMT0.05. The right panels are for RMT0.2. Red dashed lines represent equality of the two measurements. MSO, maximum stimulator output; RMT, resting motor threshold. (For interpretation of the references to colour in this figure legend, the reader is referred to the web version of this article.)

Scatter plots of the final estimates of RMT0.05 and RMT0.2 after 30 trials are presented in Fig. 2. Within-day comparisons (Session 1 vs. Session 2) demonstrated high correlation (*r* = 0.94 for both RMT0.05 and RMT0.2 conditions), whereas between-day comparisons (Session 1 vs. Session 3) showed moderate correlation (*r* = 0.61–0.63). The reproducibility was assessed using Bland-Altman analysis (Fig. 3). For the within-day comparison (Session 1 vs Session 2), the mean differences were small for both RMT0.05 (2.96 ± 4.00 % MSO) and RMT0.2 (2.21 ± 4.43 % MSO), indicating good agreement between the two sessions. The limits of agreement (mean ± 1.96 SD) ranged from − 4.89 to 10.81 % MSO for RMT0.05 and from − 6.48 to 10.89 % MSO for RMT0.2. In contrast, for the between-day comparison (Session 1 vs Session 3), the mean differences remained small (− 1.66 ± 8.99 % MSO for RMT0.05; − 2.46 ± 11.43 % MSO for RMT0.2), but the limits of agreement were wider (− 19.28 to 15.96 % MSO and − 24.86 to 19.95 % MSO, respectively), suggesting greater variability across days.

### 3.2 Sequential precision and accuracy

**Fig. 4:**
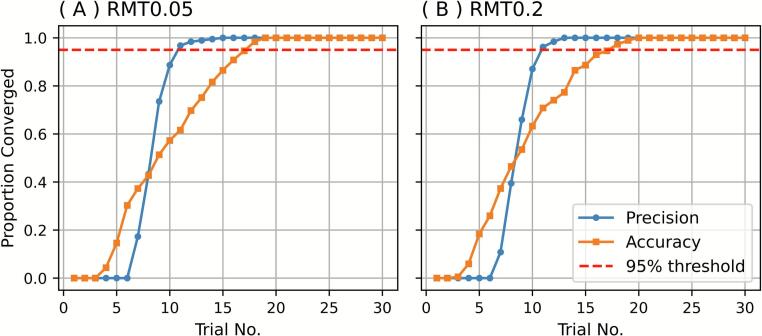
Convergence of (A) RMT0.05 and (b) RMT0.2 based on precision and accuracy. The X-axis shows the trial number (1–30). The Y-axis shows the proportion of sessions that met precision (blue) or accuracy (orange) criteria among all 185 sessions. Red dashed lines are on the 95% level. RMT, resting motor threshold. (For interpretation of the references to colour in this figure legend, the reader is referred to the web version of this article.)

Results obtained from all 185 sessions were pooled to assess the convergence of the adaptive estimates (Fig. 4). The precision criterion was satisfied in more than 95 % of sessions by the 11th trial, whereas the accuracy criterion required up to 18 trials to be met under each of the RMT0.05 and RMT0.2 conditions. These findings suggest that, although stability of estimates (precision) is achieved early, a greater number of trials must be conducted to ensure closeness to the reference threshold (accuracy).

### 3.3 Metrics of reliability

**Fig. 5:**
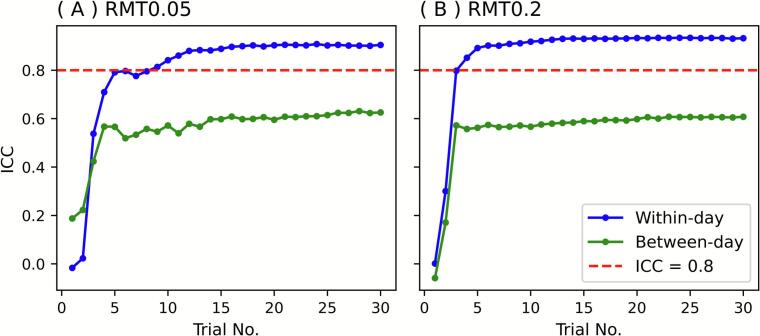
ICC for (A) RMT0.05 and (B) RMT0.2. The X-axes show the trial number (1–30). The Y-axes show ICC (2, 1). The blue line shows within-day comparison; the green line shows between-day comparison. The red dashed line represents ICC = 0.8. ICC, intraclass correlation coefficient; RMT, resting motor threshold. (For interpretation of the references to colour in this figure legend, the reader is referred to the web version of this article.)

**Fig. 6:**
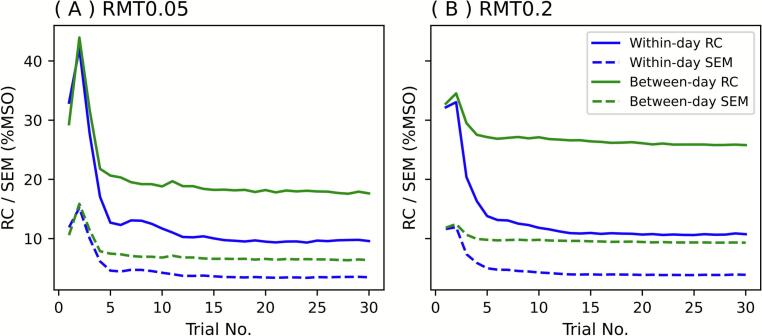
RC and SEM for (A) RMT0.05 and (B) RMT0.2. The X-axes show the trial number (1–30). The Y-axes show RC or SEM. Solid lines are RC; dashed lines are SEM. The blue line shows within-day comparison; the green line shows between-day comparison. RC, reproducibility coefficient; RMT, resting motor threshold; SEM, standard error of measurement. (For interpretation of the references to colour in this figure legend, the reader is referred to the web version of this article.)

Repeatability was assessed using ICC(2,1), RC, and SEM on both a within-day (Session 1 vs. Session 2) and a between-day (Session 1 vs. Session 3) basis. The within-day ICC rose steeply. It exceeded 0.8 as early as the ninth trial for RMT0.05 and the fourth trial for RMT0.2, whereas the between-day ICC remained lower but reached stable values thereafter (Fig. 5). Each of RC and SEM initially showed a transient increase, reflecting the wide search range of the PEST-MLE algorithm in the early phase (Fig. 6). Both indices then decreased rapidly and plateaued after approximately 15 trials, reflecting stabilization of the estimates. It is noteworthy that, by the later trials, RC converged to around 10 %MSO and SEM to below 5 %MSO for the within-day comparisons. RC values of approximately 10 %MSO indicate that repeated measurements differed by ≤10 %MSO in 95 % of cases. Correspondingly, SEM values of less than 5 %MSO suggest that the expected measurement error for a single observation was less than 5 %MSO, thereby providing an estimate of the precision of individual threshold determinations. These results suggest acceptable reproducibility for practical use.

### 3.4 Validity of the final estimate as RMT

**Fig. 7:**
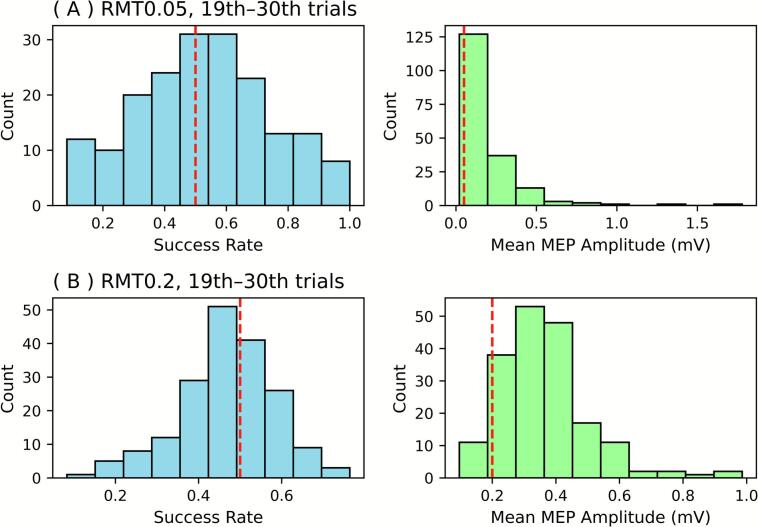
Success rates and MEP amplitudes for later sessions. For the success rate, the probability of positive MEP was averaged from the 19th to 30th trial in each session. Then histograms were created for RMT0.05 (A) and RMT 0.2 (B). The mean MEP amplitude is shown similarly.

When considering the later phase of measurements (trials 19–30), success rates were centered on 0.5 for each of RMT0.05 and RMT0.2 (Fig. 7). Similarly, the mean MEP amplitudes for these trials were slightly above the predefined cutoff of 0.05 mV and 0.2 mV. These findings suggest that the adaptive estimates had converged sufficiently by the 18th trial to fulfill the conventional definition of motor threshold, i.e., the minimum intensity eliciting MEPs above 0.05 mV or 0.2 mV in more than half of the trials.

## 4 Discussion

For this study, we implemented an adaptive threshold hunting method using the PEST-MLE algorithm to estimate the RMT for TMS-elicited MEPs. For both target amplitudes of 0.05 mV and 0.2 mV, RMT was determined reliably within approximately 18 trials. Analyses of convergence demonstrated that stability of estimates (precision) was achieved earlier than closeness to the reference value (accuracy), and that within-day repeatability was superior to between-day reproducibility. These findings suggest that the PEST-MLE algorithm provides a practical and efficient approach for accurate RMT estimation in healthy adults.

### 4.1 PEST-MLE provides rapid, reliable RMT

A key strength of the present study lies in the application of the PEST-MLE algorithm, which enables rapid and reliable estimation of RMT without requiring *a priori* knowledge of the approximate threshold. In contrast to “threshold-tracking” approaches using Qtrac software (Institute of Neurology, University College London, London, UK; distributed by Digitimer Ltd.; Welwyn Garden City, UK) (Samusyte et al., 2018, Tankisi et al., 2022, Tankisi et al., 2021, Vucic et al., 2006), which typically start from intensities close to the expected RMT such as 106 % RMT, the PEST-MLE method was able to converge to valid estimates within approximately 18 trials, even when starting from less informed initial values. Additionally, we evaluated this method systematically using multiple indices of reliability, including ICC (2, 1), RC, and SEM, as well as explicitly distinguishing precision (stability of estimates) and accuracy (closeness to a reference value). This multidimensional assessment, together with a large dataset including data of 185 sessions from 53 healthy adults, provides robust evidence that the PEST-MLE algorithm offers a practical and efficient solution for RMT estimation in both clinical and research contexts.

It could be argued that hotspot localization generally involves delivering stimuli at slightly suprathreshold intensity, thereby providing some impression of the underlying threshold. This information might be incorporated into more efficient RMT estimation. Indeed, the Qtrac-based threshold tracking approach has been shown to reach stable estimates with fewer stimuli per point (Tankisi et al., 2022). However, direct comparison with the present PEST-MLE procedure is not straightforward because the two methods differ in their initial conditions and convergence criteria. In our study, the stimulation intensity was initialized randomly between 20 % and 60 % MSO to evaluate unbiased convergence, whereas the Qtrac studies typically began near the expected RMT. The step of hot spot determination, however, is intended primarily to ensure a stable site of activation and to ensure the presence of recordable MEPs, rather than to determine RMT itself. The present study specifically addressed the subsequent estimation process in an unbiased manner and demonstrated that the PEST-MLE algorithm yielded precise, accurate, and reliable RMT values, while obviating any *a priori* assumption. Consequently, hotspot localization might introduce a practical sense of the likely threshold range, but the adaptive estimation constitutes an important and practical approach for defining RMT according to the conventional standard. For example, following an intervention, the hotspot might remain unchanged while the RMT shifts. Under such circumstances, reliance on *a priori* knowledge of the RMT might be misleading or might introduce bias. Altogether, future studies on whether and how much *a priori* knowledge of the RMT would be useful are warranted.

The analysis described herein revealed that, by the 18th trial, the threshold estimates had converged to the reference value for 95 % of participants. During the later trials (19th–30th), the average success rates were distributed around 0.5. Moreover, the mean MEP amplitudes stabilized at just above the 0.05 or 0.2 mV cutoff, which is consistent with the right-skewed distribution of MEP amplitude at a fixed intensity (Ammann et al., 2020, Goetz et al., 2014, Spampinato et al., 2023). These observations support the notion that the estimate obtained at the 18th trial can be regarded as the RMT under the conventional definition (Rossini et al., 1994). This value was consistent with earlier reports (Ah Sen et al., 2017, Awiszus, 2011, Awiszus, 2003) indicating that fewer trials are needed with the PEST-MLE method than with the conventional relative frequency method. Importantly, this convergence was observed for both RMT0.05 and RMT0.2, suggesting that the method provides consistent estimates across different baseline intensity settings.

### 4.2 Within-day and between-day comparisons

This study revealed within-day reliability as high but the between-day comparison reliability as lower, which findings accord with those of an earlier study (Samusyte et al., 2018). These findings are attributable to technical aspects such as subtle differences in coil positioning. Another potentially important factor might be the difference in the participants’ states. Although RMT might be regarded as a fundamental index that is not very susceptible to the brain state of the participant, different factors including fatigue can affect MEP amplitude and RMT (Bell et al., 2018, Kuppuswamy et al., 2015, Morris and Christie, 2020). Furthermore, variation might not simply be noise: it might include some information (Goldsworthy et al., 2021) that is indicative of some day-to-day changes of the brain state. Future studies must be conducted to elucidate the specific influences of these parameters on RMT as estimated using PEST-MLE.

### 4.3 Limitations

The rapid reduction of step size in the PEST-MLE algorithm might have led to early fulfillment of stability criteria (precision) before the estimate was sufficiently close to the true threshold (accuracy). This phenomenon, probably accentuated by the discrete 1 %MSO resolution of most stimulators, represents a methodological limitation. With PEST-MLE, the step size between successive intensity recommendations naturally decreases over the course of the procedure. As a result, small changes between updates, a marker of “precision” in this study, may be reached even when the estimate has not yet fully converged to the true threshold. Accordingly, practical implementations of ML-PEST may require an additional convergence check rather than relying solely on pulse count. In the present study, accuracy provided this additional criterion. The use of the Levenberg–Marquardt algorithm for minimizing the cost function also contributes to rapid convergence but is also sensitive to initial estimates, potentially favoring premature stabilization (Sun et al., 2022). To address these issues, several algorithmic refinements have been proposed. For example, an earlier study introduced a “roll-back” procedure when consecutive successes or failures occurred (Meincke et al., 2016). Other potential refinements include imposition of a minimum step size until a certain number of trials are completed or adopting more sophisticated approaches such as Bayesian adaptive methods (Qi et al., 2011), with some caution for its theoretical background (Awiszus, 2011) to balance precision and accuracy. These directions warrant further investigation in future studies.

Technically speaking, implementation with neuronavigation, a robot supporting coil positioning, and other sophisticated devices would increase reproducibility of the PEST-MLE if the costs are acceptable. Utility of these devices should be investigated in future studies. In this study, we fixed the session order so that the RMT0.05 was determined first in each day; this might have produced some systematic bias. Also, we relied on a rather heuristic way of determining the hot spot. Refinement in this regard can further improve the quality of measurements.

## 5 Conclusions

In conclusion, estimation of RMT using the PEST-MLE algorithm is a rapid, precise, and reliable procedure, with accuracy ensured within approximately 18 trials. The approach can be implemented readily through scripting in commonly used measurement software, making it accessible to institutes with similar apparatus for experimentation. Future studies should be conducted to evaluate its performance in real-world clinical and research contexts, including patient populations, paired-pulse paradigms, and therapeutic applications of TMS.
